# Overdose Prevention Centers and Neighborhood Commercial Activity in New York City

**DOI:** 10.1001/jamanetworkopen.2025.59863

**Published:** 2026-02-27

**Authors:** Bennett Allen, Cale Basaraba, Laura C. Chambers, Czarina N. Behrends, Brandon D. L. Marshall, Magdalena Cerdá

**Affiliations:** 1 Center for Opioid Epidemiology and Policy, Department of Population Health, New York University Grossman School of Medicine, New York; 2 Department of Epidemiology, Brown University School of Public Health, Providence, Rhode Island; 3 Department of Population Health Sciences, Weill Cornell Medicine, New York, New York

## Abstract

**Importance:**

Overdose prevention centers (OPCs) are interventions to reduce overdose mortality and support health care engagement. In the US, concerns have been raised that OPCs may be associated with reduced economic activity in their surrounding neighborhoods.

**Objective:**

To evaluate changes in the local economic activity in New York City (NYC), measured by neighborhood-level foot traffic and consumer spending, following the opening of the first 2 publicly recognized OPCs in the US.

**Design, Setting, and Participants:**

This cohort study used anonymized mobility and spending data from June 1, 2021, to June 13, 2022, for the areas surrounding the East Harlem and Washington Heights OPCs in NYC. These neighborhoods were defined using 5-minute and 10-minute walking buffers and Business Improvement Districts (BIDs). Synthetic control donors included walking buffers and BIDs around syringe service programs without OPCs and opioid treatment programs that were operational as of OPCs’ opening. Analyses were conducted from February to July 2025.

**Exposures:**

Opening of the 2 NYC OPCs on November 30, 2021.

**Main Outcomes and Measures:**

Primary outcomes were foot traffic and in-person consumer spending within 10-minute walking buffers. Secondary analyses considered 5-minute walking buffers and BIDs. Augmented synthetic control models were adjusted for neighborhood-level demographic and socioeconomic features, with fit assessed using root mean squared error before OPC opening. Permutation tests and conformal inference were used to assess significance.

**Results:**

A total of 27 biweekly observations (13 in pre-OPC and 14 in post-OPC periods) were analyzed. The 10-minute walking buffer analyses captured 1259 consumer spending sites and 7816 foot traffic sites across 2 treated buffers and 56 donor buffers. In East Harlem, the average treatment effect on the treated (ATT) estimate (SE) was –$21.96 ($40.53) for consumer spending (*P* = .16) and 1.28 (5.40) visits for foot traffic (*P* = .19). In Washington Heights, ATT (SE) estimates were $14.94 ($37.38) for consumer spending (*P* = .13) and 0.44 (3.54) visits for foot traffic (*P* = .97). Secondary analyses produced consistent results. No statistically significant results were observed at any post-OPC time point.

**Conclusions and Relevance:**

This cohort study found that OPC opening was not associated with significant changes in local economic activity. Given the absence of observed economic harms, policy debates should instead focus on the public health implications of OPCs.

## Key Points

**Question:**

Are overdose prevention centers (OPCs) associated with neighborhood economic activity?

**Findings:**

In this cohort study of the first 2 publicly recognized OPCs in the US, there were no significant changes in foot traffic and consumer spending after the OPCs opened in the East Harlem and Washington Heights neighborhoods in New York City.

**Meaning:**

The finding suggests that, given the absence of observed economic harms, policy debates should instead focus on the public health implications of OPCs.

## Teaser

This cohort study assesses whether the opening of the first publicly recognized overdose prevention centers in the US was associated with changes in foot traffic and consumer spending at local businesses.

## Introduction

Unintentional drug overdose is a leading cause of preventable death in the US, with rapid increases since 2020 driven by illicitly manufactured synthetic opioids and the COVID-19 pandemic.^1,2^ Despite a considerable decrease from 2023 to 2024, overdose deaths remain at epidemic levels.^3^ In response, public health policy has increasingly emphasized harm reduction strategies, which aim to mitigate overdose mortality and morbidity by providing access to health care, treatment, and social supports without requiring abstinence.^4^

Among harm reduction responses, overdose prevention centers (OPCs) have gained considerable attention. OPCs are designed to prevent overdose deaths by providing a hygienic space where people can be monitored following drug use, access sterile equipment, and receive medical intervention in the case of overdose^5,6^ OPCs serve as entry points to substance use disorder treatment, primary and psychiatric health care, and social services for the populations they serve.^7^ More than 200 OPCs exist in Europe, Canada, and Australia.^8^ However, only 3 publicly recognized OPCs exist in the US: 2 in New York City (NYC), which opened in November 2021,^9^ and 1 in Providence, Rhode Island, which opened in January 2025.^10^

At the neighborhood level, OPCs have been associated with decreases in overdose mortality,^11,12^ fewer emergency service calls for overdose response,^13^ and reductions in drug-related crime.^14^ Despite these public health and community benefits, political and public opposition to OPCs is common.^15^ Critics often cite concerns about neighborhood safety; disorder; and cumulative siting burden, including potential adverse economic consequences for residents and local businesses.^16^ Concerns about adverse outcomes for local businesses have been particularly prominent in NYC,^17,18,19^ although early evidence suggests that the NYC OPCs are not associated with increased neighborhood crime or disorder.^14,20^

However, empirical evidence on such neighborhood-level economic factors remains limited. Studies in Montreal, Canada, and Sydney, Australia, found that OPCs may have been associated with small, short-term disruptions to real estate markets in their immediate vicinity^21,22^; however, to our knowledge, no studies have examined the implications for consumer spending or foot traffic, and none has evaluated area-level economic outcomes in the US. As jurisdictions across the US seek to open OPCs, evidence about their broader economic impacts, if any, is urgently needed.

Our study addresses this gap. We aimed to evaluate changes in the local economic activity in NYC, measured by neighborhood-level foot traffic and consumer spending, following the opening of the first 2 publicly recognized OPCs in the US. The NYC OPCs are embedded in the syringe service program (SSP) OnPoint NYC, which operates 2 storefront locations: OnPoint in East Harlem and OnPoint in Washington Heights.^9^ These SSPs provide a range of harm reduction services, including syringe access and disposal, overdose education, naloxone distribution, primary care, infectious disease testing and treatment, and mental health and social support services. On November 30, 2021, OnPoint NYC added OPC services to both storefront locations, representing the first publicly recognized supervised drug consumption spaces in the country.^5^ In more recent years, policy plans to open additional OPCs across NYC have faced considerable public and political opposition.^23,24^

As a densely populated and highly walkable city, NYC represents an ideal setting to assess the association between OPCs and neighborhood consumer activity.^25^ By providing the first empirical evidence of this association, findings of this study could inform policymakers, business owners, and community stakeholders engaged in discussions regarding harm reduction strategies and urban economic development.

## Methods

### Study Design

We conducted a retrospective cohort study of the NYC OPCs in East Harlem and Washington Heights. Using anonymized mobility and consumer spending data, we applied augmented synthetic control (ASC) models to compare observed post-OPC implementation outcomes with counterfactuals constructed from comparable areas without OPCs. The NYU Langone Health Institutional Review Board deemed this study exempt from ethics review and the informed consent requirement because it did not involve human participants. We followed the Strengthening the Reporting of Observational Studies in Epidemiology (STROBE) reporting guideline.^26^

### Data Source

We obtained data from SafeGraph, a geolocation analytics firm.^27^ SafeGraph maintains a database of more than 53 million locations across the US. We used 2 linked SafeGraph data types: (1) geolocated mobility data from points of interest (POI), which capture movement into commercial storefronts, airports, restaurants, and medical offices, and (2) consumer credit card spending data from a subset of these POIs. Because SafeGraph spending data are available for a subset of POIs, the number of consumer spending POIs was smaller than the number of foot traffic POIs. A detailed description of the SafeGraph data sources is provided in the eMethods in Supplement 1.

### Study Period

Our study period was from June 1, 2021, to June 13, 2022. This period was split into 13 biweekly observations in the preintervention (hereafter pre-OPC) period from June 1, 2021, to November 29, 2021, and 14 biweekly observations in the postintervention (hereafter post-OPC) period from November 30, 2021, to June 13, 2022. We selected this period to ensure sufficient pre-OPC coverage during a time of relative stability following NYC’s initial COVID-19 lockdowns, while also capturing the immediate post-OPC patterns during the first 6 months of OPC operations, the period for which any economic impacts would be anticipated as most acute. We did not extend the pre-OPC period further due to volatility in consumer mobility and spending during the early stages of the COVID-19 pandemic in NYC.^28^ We did not extend the post-OPC period given data availability for the pre-OPC period.

### Study Setting

**Figure 1.**
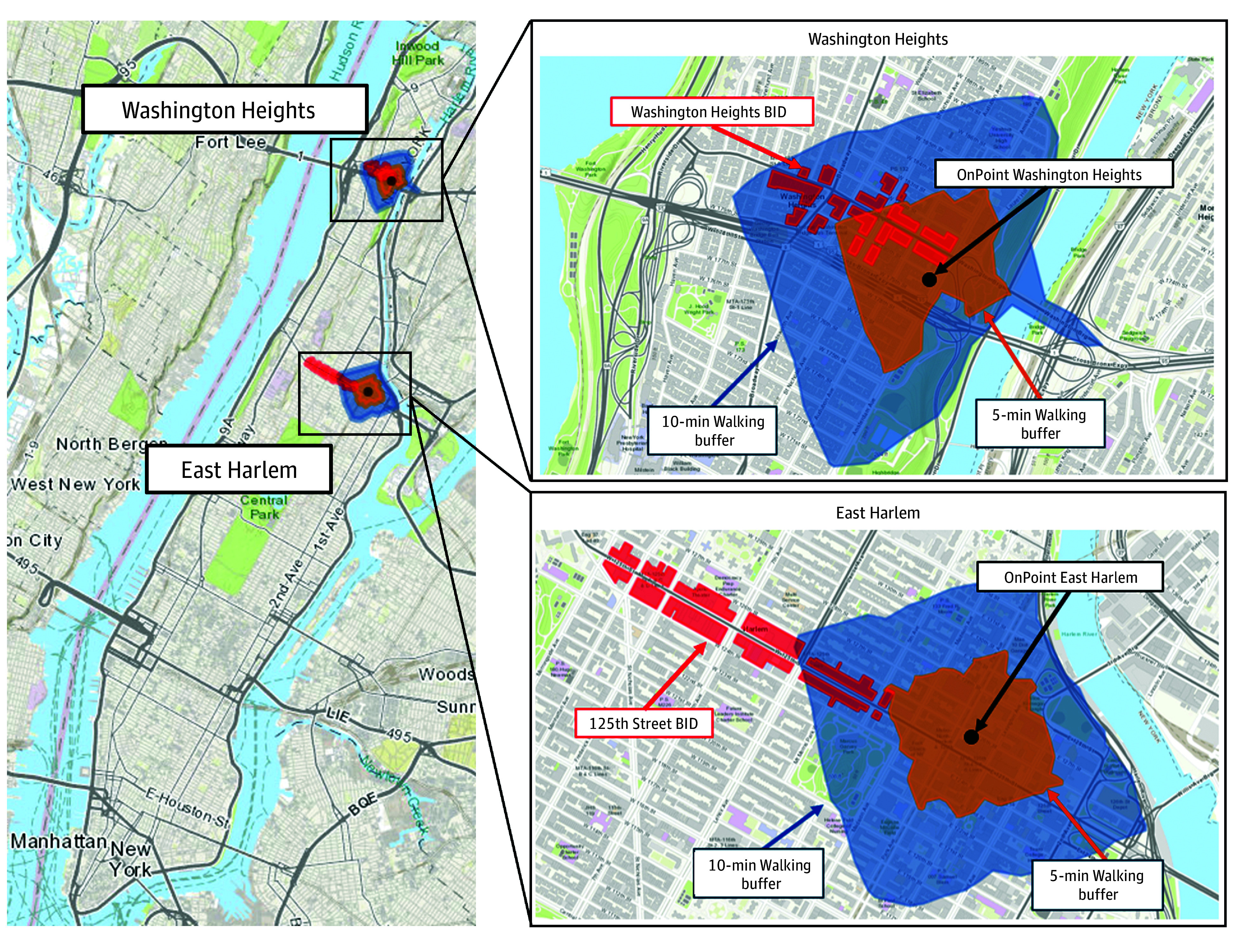
Map of OnPoint East Harlem and OnPoint Washington Heights Locations With Buffer and Business Improvement District (BID) Analysis Areas

We defined the neighborhoods surrounding the OPCs in 3 ways: (1) 10-minute walking buffers; (2) 5-minute walking buffers; and (3) Business Improvement Districts (BIDs), which are areas in NYC where businesses collaborate to enhance neighborhood economic vitality (Figure 1). To create walking buffers around sites, we queried the publicly available OpenStreetMap (OpenStreetMap Foundation) routing service with the osrm package, using the standard walk speed of 5 km/h.^29^

### Exposure and Outcomes

The exposure of interest was the publicly and politically acknowledged opening of the NYC OPCs on November 30, 2021. This exposure reflects the addition of OPC services within the existing OnPoint NYC service infrastructure on this date.

Primary outcomes were foot traffic and in-person consumer spending within 10-minute walking buffers. Secondary analyses considered 5-minute walking buffers and BIDs. For each POI with complete foot traffic or consumer spending data in our defined neighborhoods, we aggregated all foot traffic visits or in-person consumer spending into biweekly periods and then, due to the observed right-skew in both outcomes, we calculated the median as the measure of central tendency across POIs in each catchment area. To adjust for inflation during the study period, we set all consumer spending to June 2022 dollars using the monthly Consumer Price Index for all urban consumers from the Federal Reserve Economic Data.^30^ Only POIs with valid nonmissing foot traffic or consumer spending data throughout the entire study period were used in the analysis; the small number of POIs (<1%) with negative spending values (likely due to data entry or processing errors) were removed. Given the distinct socioeconomic and demographic profiles of East Harlem and Washington Heights and the geographic distance between neighborhoods, we estimated OPC impacts separately for the 2 neighborhoods.^20^

### Covariates

We included several potential neighborhood-level factors in foot traffic and consumer spending in our models: neighborhood socioeconomic conditions derived from the US Census Bureau American Community Survey, POI-level variables, COVID-19 hospitalization rates,^31^ and NYPD arrests.^32^ Full details on these covariates are provided in the eMethods in Supplement 1. An overview of the data sources used in this study is presented in eTable 1 in Supplement 1.

### Statistical Analysis

We calculated the median and IQR of biweekly median consumer spending and foot traffic for each catchment area (10-minute walk, 5-minute walk, and BID) in the East Harlem and Washington Heights OPC neighborhoods and synthetic donor units, stratified by pre-OPC and post-OPC periods. Differences between periods were assessed using the Wilcoxon rank sum test. Two-sided *P* < .05 indicated statistical significance.

We used the ASC method^33^ to examine the association of opening the NYC OPCs with median biweekly consumer spending and foot traffic in their surrounding neighborhoods. Synthetic control methods are used to estimate associations of interventions that occur in a small number of treated units, often states or administrative areas, when there is no appropriate control for comparison. We tested for significance with placebo testing, assessed model fit using root mean squared error (RMSE), and used a conformal inference procedure to construct 95% CIs and evaluate the significance of associations at post-OPC time points. Full details of the statistical analysis are described in the eMethods in Supplement 1.

For the primary analysis, we separately evaluated the association of OPC openings in East Harlem and in Washington Heights with changes in consumer spending and foot traffic in the 10-minute and 5-minute walking buffers around each site. We considered 10-minute walking buffers around SSPs without OPC services and opioid treatment programs (OTPs) as our donor pool. We also evaluated the association of the East Harlem OPC opening for the 125th Street BID and the association of Washington Heights OPC opening for the 181st Street BID, using all other NYC BIDs (n = 74) as the donor pool to construct each synthetic control; none of these donor BIDs were within the same community districts as the treated BIDs. Full details of the donor selection process are provided in the eMethods in Supplement 1, and the numbers of POIs within donor units for each area site and buffer analysis are presented in Table 1. Analyses and visualizations were performed between February and July 2025 using R, version 4.4 (R Project for Statistical Computing).^34^

**Table 1.** Consumer Spending and Foot Traffic in the Neighborhoods Surrounding Overdose Prevention Centers in New York City

	**POI No.**	Median (IQR)			***P* value^a^**
		**Overall**	**Pre-OPC period^b^**	**Post-OPC period^c^**	
**Consumer spending, $**					
10-min Walking buffer					
East Harlem	11	198.12 (181.06-233.75)	204.72 (181.38-239.96)	195.25 (173.46-233.06)	.52
Washington Heights	21	255.55 (204.93-286.61)	278.82 (239.41-286.61)	228.99 (173.02-280.57)	.11
All donors	1227	280.24 (152.14-441.83)	327.98 (174.11-467.63)	241.01 (132.61-403.37)	<.001
BID					
East Harlem	41	493.43 (393.57-631.19)	594.02 (397.29-631.19)	490.61 (348.78-556.37)	.26
Washington Heights	15	193.60 (147.02-248.14)	216.91 (192.30-283.94)	164.75 (138.92-200.97)	.03
All donors	3111	323.22 (184.60-540.60)	371.92 (220.52-589.12)	274.18 (152.71-482.41)	<.001
5-min Walking buffer					
East Harlem	5	172.16 (121.37-200.65)	178.58 (161.76-200.65)	137.49 (108.26-180.47)	.11
Washington Heights	3	77.00 (49.57-105.85)	80.69 (47.11-150.83)	74.74 (57.15-98.27)	.79
All donors	397 In donors for East Harlem; 374 in donors for Washington Heights	323.93 (175.73-568.66)	383.15 (206.96-642.79)	283.84 (146.77-497.70)	<.001
**Foot traffic, visits**					
10-min Walking buffer					
East Harlem	156^d^	67.5 (57.5-84.5)	61.5 (56.5-68.5)	77.3 (67.0-93.5)	.03
Washington Heights	159^d^	54.0 (49.0-66.0)	52.0 (49.0-54.0)	63.0 (51.0-71.0)	.04
All donors	7501^d^	61.0 (45.0-96.0)	54.5 (42.0-86.0)	66.0 (48.0-103.5)	<.001
BID					
East Harlem	82^d^	66.5 (62.0-85.0)	64.5 (62.5-68.5)	76.8 (62.0-93.0)	.17
Washington Heights	45^d^	46.0 (41.0-62.0)	44.0 (40.0-45.0)	56.0 (46.0-65.0)	.003
All donors	8231^d^	69.0 (41.0-154.0)	63.5 (37.0-151.0)	73.0 (46.0-155.8)	<.001
5-min Walking buffer					
East Harlem	54^d^	97.0 (76.0-124.0)	77.5 (74.5-84.5)	117.8 (101.0-129.5)	<.001
Washington Heights	41^d^	63.0 (58.0-71.0)	61.0 (58.0-63.0)	69.5 (58.0-78.0)	.11
All donors	2478^d^	64.0 (44.0-115.0)	58.0 (41.5-105.0)	70.5 (47.5-123.0)	<.001

Abbreviations: BID, Business Improvement District; OPC, overdose prevention center; POI, point of interest.

^a^ *P* values were derived from Wilcoxon rank sum test, which calculated the differences between exposure periods.

^b^ Pre-OPC period spanned June 1, 2021, to November 29, 2021, and consisted of 13 observations.

^c^ Post-OPC period spanned November 30, 2021, to June 13, 2022, and consisted of 14 observations.

^d^ POI counts reflect the number of eligible POIs contributing to the catchment area medians and were restricted to POIs with complete valid consumer spending or foot traffic observations across the full study period. Counts differ for consumer spending and foot traffic because spending data were available for a subset of POIs.

## Results

### Descriptive Analysis

Descriptive findings are displayed in Table 1. Across 27 biweekly observations spanning June 1, 2021, to June 13, 2022 (13 observations in pre-OPC and 14 observations in post-OPC periods), the primary 10-minute walking buffer analyses included 315 foot traffic POIs (156 in East Harlem and 159 in Washington Heights) and 32 consumer spending POIs (11 in East Harlem and 21 in Washington Heights). The donor pool comprised 56 walking buffers around SSPs without OPC services and OTPs (16 SSPs and 40 OTPs for foot traffic; 16 SSPs and 39 OTPs for consumer spending), including 7501 foot traffic POIs and 1227 consumer spending POIs.

Biweekly median consumer spending and foot traffic are displayed in Table 1. There were significant decreases in median (IQR) biweekly consumer spending in all donor units from the pre-OPC to post-OPC periods across all catchment areas (eg, 10-minute walking buffer: $327.98 [$174.11-$467.63] to $241.01 [$132.61-$403.37]; *P* < .001) as well as a significant decrease in median (IQR) consumer spending in the Washington Heights BID ($216.91 [$192.30-$283.94] to $164.75 [$138.92-$200.97]; *P* = .03). For median (IQR) biweekly foot traffic, there were significant increases in all donor units from the pre-OPC to post-OPC periods across all area definitions (eg, 5-minute walking buffer: 58.0 [41.5-105.0] visits to 70.5 [47.5-123.0] visits; *P* < .001), significant increases in Washington Heights across both 10-minute walking buffer (52.0 [49.0-54.0] visits to 63.0 [51.0-71.0] visits; *P* = .04) and BID (44.0 [40.0-45.0] visits to 56.0 [46.0-65.0] visits; *P* = .003), and a significant increase in the 10-minute (61.5 [56.5-68.5] visits to 77.3 [67.0-93.5] visits; *P* = .03) and 5-minute (77.5 [74.5-84.5] visits to 117.8 [101.0-129.5] visits; *P* < .001) walking buffers around the East Harlem OPC. eFigure 5 in Supplement 1 shows log median biweekly consumer spending and foot traffic across the study period for all NYC Census tracts.

### Augmented Synthetic Control Models

**Figure 2.**
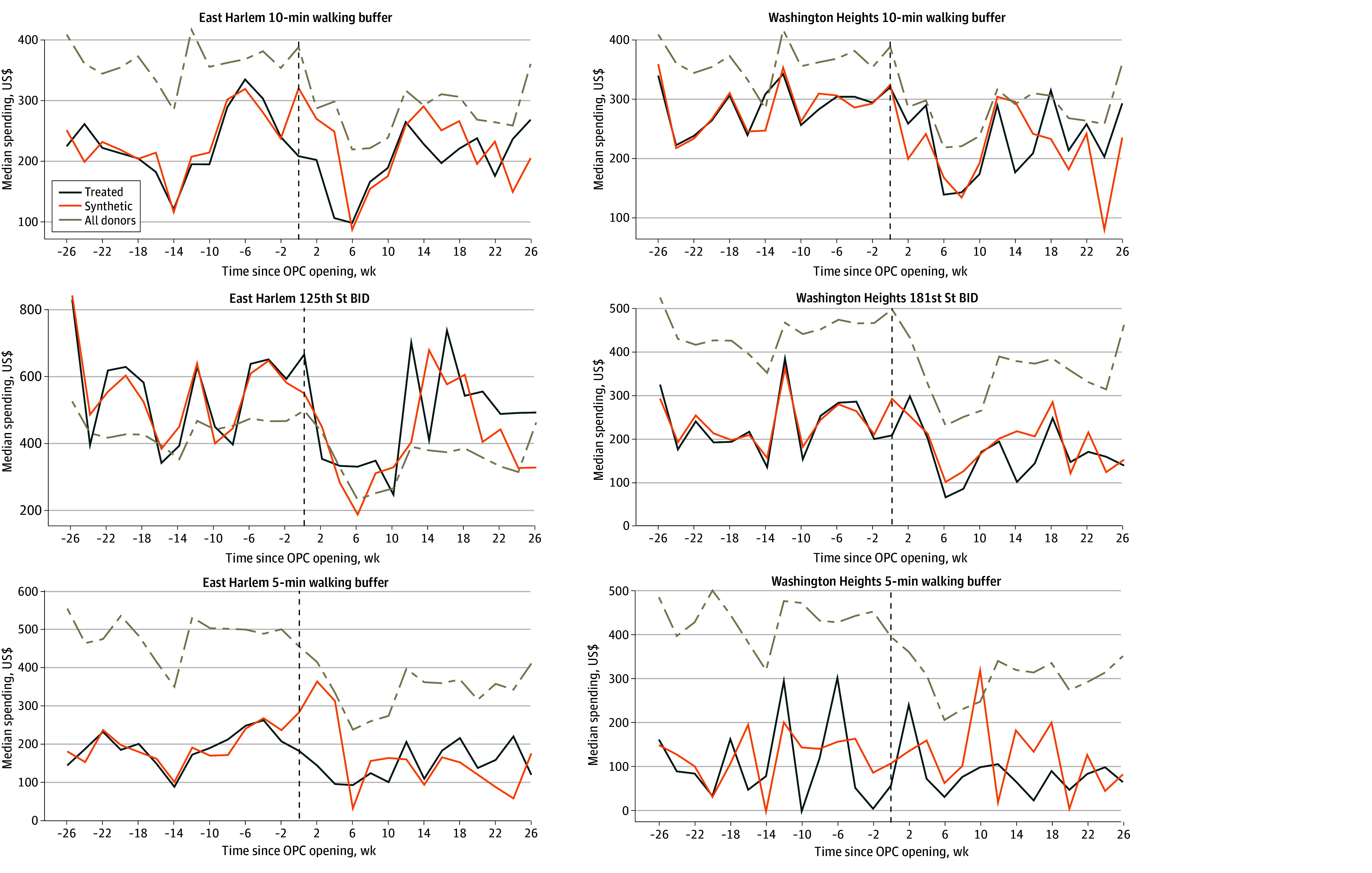
Line Graphs of Consumer Spending for Treated and Synthetic Control Neighborhoods From Augmented Synthetic Control Models With Neighborhood Covariates Neighborhood covariates were median age, median household income, proportion of residents unemployed, proportion of families living below the federal poverty line, proportion of individuals 25 years or older with a high school diploma, gentrification, proportion of grocery store points of interest (POIs), proportion of restaurant POIs, COVID-19 hospitalization rate, and New York Police Department arrests. BID indicates Business Improvement District; OPC, overdose prevention centers.

**Figure 3.**
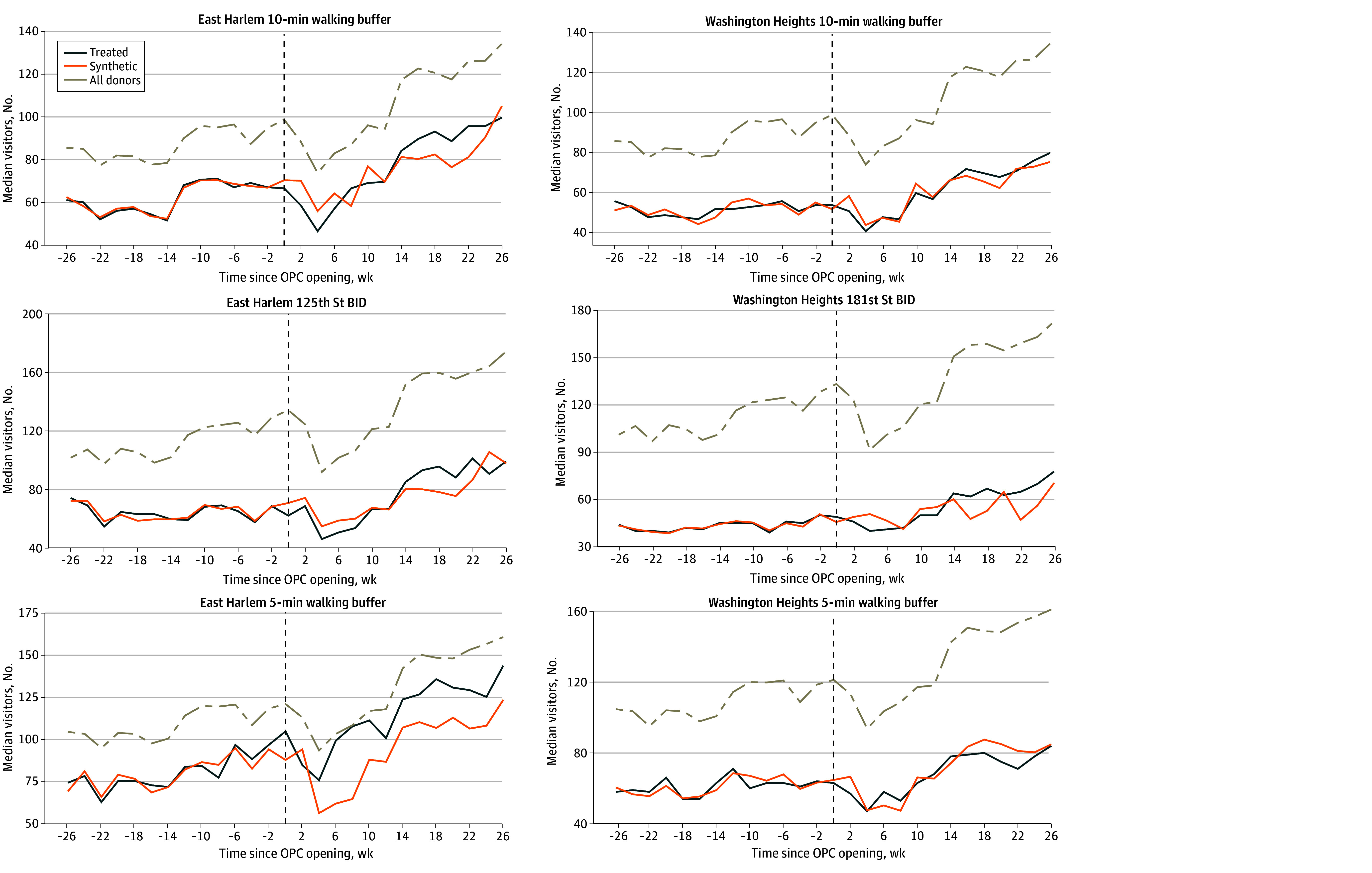
Line Graphs of Foot Traffic for Treated and Synthetic Control Neighborhoods From Augmented Synthetic Control Models With Neighborhood Covariates Neighborhood covariates were median age, median household income, proportion of residents unemployed, proportion of families living below the federal poverty line, proportion of individuals 25 years or older with a high school diploma, gentrification, proportion of grocery store points of interest (POIs), proportion of restaurant POIs, COVID-19 hospitalization rate, and New York Police Department arrests. BID indicates Business Improvement District; OPC, overdose prevention centers.

Visualizations and small RMSE values compared with observed values in the pre-OPC period indicated that ASC models with neighborhood covariates produced synthetic controls that closely fit pre-OPC biweekly median foot traffic and consumer spending patterns across all area definitions (Figures 2 and 3, Table 2). Similarly well-fitted synthetic controls were produced for ASC models without covariates (eFigures 2-3 and eTable 2 in Supplement 1).

**Table 2.** Augmented Synthetic Control Results for Consumer Spending and Foot Traffic With Neighborhood Covariates^a^

	RMSE		Post-OPC to Pre-OPC ratio	ATT (SE)^b^	*P* value^c^
	Pre-OPC period	Post-OPC period			
**Consumer spending**					
10-min Walking buffer					
East Harlem	23.6945	67.6185	2.8538	−21.96 (40.53)	.16
Washington Heights	20.4358	59.0602	2.8900	14.94 (37.38)	.13
BID					
East Harlem	46.1093	152.6404	3.3104	59.07 (55.36)	.27
Washington Heights	18.5965	50.2025	2.6996	−24.46 (31.54)	.36
5-min Walking buffer					
East Harlem	22.9796	105.7257	4.6009	−16.87 (152.40)	.12
Washington Heights	88.9465	93.4593	1.0507	−37.09 (89.45)	.83
**Foot traffic**					
10-min Walking buffer					
East Harlem	1.1095	8.6087	7.7590	1.28 (5.40)	.19
Washington Heights	2.6505	3.6193	1.3655	0.44 (3.54)	.96
BID					
East Harlem	2.5216	9.9380	3.9411	0.79 (11.80)	.61
Washington Heights	1.0045	9.2623	9.2208	3.21 (11.63)	.77
5-min Walking buffer					
East Harlem	3.7848	23.4435	6.1941	20.39 (9.51)	.14
Washington Heights	3.2920	5.9847	1.8180	−2.19 (6.75)	.89

Abbreviations: ATT, average treatment effect on the treated; BID, Business Improvement District; OPC, overdose prevention center; POI, point of interest; RMSE, root mean squared error.

^a^ Neighborhood covariates were median age, median household income, proportion of residents unemployed, proportion of families living below the federal poverty line, proportion of individuals 25 years or older with a high school diploma, gentrification, proportion of grocery store POIs, proportion of restaurant POIs, COVID-19 hospitalization rate, and New York Police Department arrests.

^b^ Jackknife SE.

^c^ Permutation *P* values from placebo tests.

### 10-Minute Walking Buffer Analysis

In our primary ASC analysis of 10-minute walking buffers that included neighborhood covariates, there was no evidence that the opening of the NYC OPCs had a significant association with median biweekly consumer spending or foot traffic over the study period (Table 2, Figure 2 and Figure 3). In East Harlem, the average treatment effect on the treated (ATT) estimate (SE) was –$21.96 ($40.53) for consumer spending (*P* = .16) and 1.28 (5.40) visits for foot traffic (*P* = .19). In Washington Heights, ATT (SE) estimates were $14.94 ($37.38) for consumer spending (*P* = .13) and 0.44 (3.54) visits for foot traffic (*P* = .97). We also did not identify any significant associations at any specific post-OPC time point when using conformal inference (eFigure 1 in Supplement 1). Similar results were obtained from ASC analyses without neighborhood covariates (eTable 2 and eFigures 2-4 in Supplement 1).

### 5-Minute Walking Buffer Analysis

There was also no evidence that the NYC OPC openings had a significant association with consumer spending or foot traffic in 5-minute walking buffers, overall or at a specific post-OPC time point (Table 2, Figures 2-3; eFigure 1 in Supplement 1). Consistent results were obtained from ASC analyses without neighborhood covariates (eTable 2 and eFigures 2-4 in Supplement 1).

### Business Improvement District Analysis

Similarly, there was no evidence that the opening of the NYC OPCs had a significant association with median biweekly consumer spending or foot traffic in BIDs near to each OPC across the post-OPC period (Table 2, Figure 2 and Figure 3). We also did not observe significant associations at any time point (eFigure 1 in Supplement 1). Similar results were obtained from ASC analyses without neighborhood covariates (eTable 2 and eFigures 2-4 in Supplement 1).

## Discussion

This study estimated the association of the NYC OPCs with local economic activity using novel, anonymized foot traffic and consumer spending data. Despite concerns that OPCs may reduce neighborhood commerce,^35^ we did not find meaningful changes in nearby foot traffic or consumer spending after the OPCs opened.

Findings of this study were consistent across model types and geographic areas, with no evidence that OPCs adversely affected local businesses. Additionally, the results align with prior studies showing no association between OPCs and neighborhood decline.^36^ While we observed concordance between walking buffer–based and BID-based analyses, BID boundaries are administratively defined and may only partially overlap with a plausible exposure catchment around each OPC. For example, portions of the 125th Street BID are several blocks from the East Harlem OPC and fall outside of a 10-minute walking buffer. If the economic consequences of OPC implementation are highly localized, such spatial mismatch may dilute BID-level estimates.^37^ However, we also used multiple walking buffers to capture proximal commercial environments, with null findings consistent across both BID- and buffer-based analyses.

These findings should be interpreted with precision and nuance regarding the operationalization of the study exposure. The 2 OPCs opened within the established OnPoint NYC infrastructure^9^; as such, the policy intervention on November 30, 2021, was the formal, publicly recognized addition of OPC services to both OnPoint NYC storefront SSP locations. Our estimates, therefore, are indicative of the neighborhood economic implications of adding and publicly authorizing OPC services within an existing harm reduction service infrastructure, rather than the implications of siting a new, stand-alone OPC in a neighborhood without existing parent harm reduction services. This finding is particularly relevant in the context of public discussion of neighborhood social and health service saturation.^38^ The noted minimal marginal effects of adding an OPC component to existing SSP sites point to the advantage of siting OPCs within existing harm reduction services, where they may serve an established community in need. That is, the addition of an OPC component to existing SSP services has the potential to address the public health consequences of substance use in areas with a long history of this problem without posing adverse economic consequences for residents. Future research should measure the economic impacts of newly sited OPC facilities, such as the OPC in Providence, Rhode Island, which opened in January 2025.^10^

We documented descriptive changes over time in the treated and donor neighborhoods: specifically, increased biweekly median foot traffic alongside decreased biweekly median consumer spending. These patterns may remain salient to local stakeholders and reflect changing economic conditions during the study period, consistent with citywide patterns in donor units. Modest increases in foot traffic likely reflect the lifting of COVID-19 restrictions and neighborhood-level racial and economic disparities in lockdown enforcement as well as concentrations of essential workers in the OPC neighborhoods.^39,40^ Concurrent modest decreases in consumer spending may reflect broader US economic conditions during the early and post–COVID-19 recovery periods: national economic growth slowed in the second half of 2021 and was negative in the first quarter of 2022.^41^ Within this context, our synthetic control analysis estimated whether observed changes exceeded what would have been expected at the neighborhood level in the absence of the OPC openings.

While the public health benefits and potential health care cost savings of OPCs are well established,^6,42^ their neighborhood economic consequences have been largely untested. By leveraging high-resolution geolocation and transaction data, this study provides the first rigorous empirical evidence of the economic impacts of OPCs.^43^ Stability of local commerce in East Harlem and Washington Heights suggests that OPCs can be implemented in high-need urban areas with minimal or no consequence to commercial activity. These findings are consistent with research documenting local business stakeholders’ support for OPCs as a novel opportunity to reduce drug use in public,^17^ which may otherwise deter consumer activity.^44^

Our results counter political rhetoric that OPCs drive economic decline.^45^ Consumer behavior did not measurably change at any point after OPC opening. Given the absence of economic harm, and consistent evidence of the individual and community health benefits of OPCs,^6,42^ policy conversations may more effectively frame OPCs as a public health intervention rather than an economic threat. With limited US research on community-level OPC impacts, our study adds critical evidence for decision-makers at the federal, state, and local levels.^46^

### Limitations

This study has several limitations. First, our study period covers only the first 6 months following OPC implementation. While this time frame allows detection of immediate economic consequences, consistent with short-term outcomes observed in Sydney, Australia,^22^ and Montreal, Canada,^21^ longer-term patterns may differ. Future studies should extend this analysis to assess whether OPCs have sustained or changed the economic activity over time. Second, while we adjusted for key neighborhood and economic conditions, unmeasured time-varying confounding factors—such as evolving business practices, neighborhood-level policy changes, or broader shifts in consumer behavior—may have affected the findings, and the ASC approach cannot account for these factors.^47^ Third, the study focused on NYC, a singularly dense city in terms of foot traffic patterns,^48^ limiting generalizability to other US contexts where the economic and social dynamics surrounding OPCs may differ. Future research should consider the economic impacts of OPCs in other jurisdictions (eg, Providence, Rhode Island) as they open across the US.^49^ Fourth, we assessed only consumer behavior factors rather than other economic indicators (eg, commercial vacancy rates, property values, and employment patterns),^50^ which may provide a more holistic picture of the neighborhood-level implications of OPCs.

Fifth, our study period included the Delta and Omicron waves of the COVID-19 pandemic,^51^ which may have independently played a role in consumer behavior across NYC. Although we adjusted for COVID-19 hospitalization rates and restricted the study period to NYC’s final reopening phase,^52^ we recognize that hospitalization rates do not fully capture all pandemic-related factors (eg, consumer voluntary risk avoidance), and our analyses may be subject to residual confounding due to these unmeasured pandemic-related factors. Sixth, due to data sparsity, we were unable to model the economic impacts for only those POIs most proximal to the OPCs (eg, ≤250 m), although we assessed several catchment areas of varying size consistent with strategies for the epidemiological evaluation of hyperlocal interventions such as OPCs.^20^ Seventh, the ASC approach assumes no interference between treated and donor units.^53^ In NYC, the SSP and OTP services operate within an interconnected ecosystem in which some redistribution of service use by clients across multiple sites is plausible.^54^ To mitigate the potential for this bias, we excluded any overlapping buffers from analysis, although some interference remains possible. Eighth, because the launch of the NYC OPCs coincided with a widely reported public announcement and local government recognition of OPCs as additions to the established neighborhood SSP infrastructure,^5^ we cannot disentangle the implications of novel OPC operations in and of themselves from contemporaneous public awareness, media attention, or policy change, nor can our findings respond to the question of newly established OPCs in the absence of existing harm reduction services and community engagement. Ninth, although limited, OPC-adjacent overdose mitigation practices (eg, staff monitoring of clients in SSP bathrooms) may have occurred at the treatment sites prior to the publicly recognized OPC launch on November 30, 2021, but such policies are not comparable to the formally implemented OPC model in terms of capacity, services delivered, or intensity and continuity of supervision.^55^

Finally, SafeGraph data were collected for commercial, nonresearch purposes and may be subject to biases in coverage and representation, including lack of spending data from businesses without a credit card or electronic sales infrastructure (eg, cash-only vendors). To ensure consistency in outcome measurement, we restricted analyses to POIs with complete and valid foot traffic or consumer spending data across the full study period, which may introduce selection bias if we differentially excluded smaller, lower-volume, or cash-only POIs. In addition, although recent research has shown that SafeGraph data may underrepresent the population in dense urban areas, with higher proportions of low-income and racially or ethnically minoritized residents compared with rural, high-income, and majority White geographies,^56^ a growing body of work has identified that SafeGraph may serve as a functional data source to ascertain a valid signal of neighborhood-level consumer behavior in urban settings, including NYC.^57,58,59^ Future research into the economic impacts of OPCs should validate our findings through direct measurement of other neighborhood economic indicators, such as business and license issuances, business openings and closures, and storefront vacancy and land use patterns.

## Conclusions

The findings of this cohort study inform ongoing policy debates by suggesting that, in this setting, the addition of OPC services was not associated with economic changes at the neighborhood level. Considering the absence of observed economic harms, policy debates should center on the public health implications of OPCs in lieu of secondary neighborhood economic outcomes.

## Notes

Supplement 1. **eFigure 1.** Estimated Effects at Post-OPC Timepoints From Augmented Synthetic Control Models With Neighborhood Covariates^1^, 95% Confidence Intervals From Conformal Inference **eFigure 2.** Consumer Spending for Treated and Synthetic Neighborhoods From Augmented Synthetic Control Models Without Neighborhood Covariates **eFigure 3.** Foot Traffic for Treated and Synthetic Neighborhoods From Augmented Synthetic Control Models Without Neighborhood Covariates **eFigure 4.** Estimated Effects at Post-OPC Timepoints From Augmented Synthetic Control Models Without Neighborhood Covariates, 95% Confidence Intervals From Conformal Inference **eFigure 5.** Census Tract Log Median Biweekly Spending and Log Median Biweekly Foot Traffic in Study Period **eTable 1.** Data Sources Used for Study Analyses **eTable 2.** Augmented Synthetic Control Results for Consumer Spending and Foot Traffic Without Neighborhood Covariates
eMethods

eReferences

Supplement 2.
Data Sharing Statement
