# Supplementary material for: Overdose Prevention Centers and Neighborhood Commercial Activity in New York City
Source: JAMA Netw Open. 2026 Feb 27;9(2):e2559863. doi: 10.1001/jamanetworkopen.2025.59863 (PMC12949448; doi:10.1001/jamanetworkopen.2025.59863)
Supplement: Supplement 1. — eFigure 1. Estimated Effects at Post-OPC Timepoints From Augmented Synthetic Control Models With Neighborhood Covariates1, 95% Confidence Intervals From Conformal Inference eFigure 2. Consumer Spending for Treated and Synthetic Neighborhoods From Augmented Synthetic Control Models Without Neighborhood Covariates eFigure 3. Foot Traffic for Treated and Synthetic Neighborhoods From Augmented Synthetic Control Models Without Neighborhood Covariates eFigure 4. Estimated Effects at Post-OPC Timepoints From Augmented Synthetic Control Models Without Neighborhood Covariates, 95% Confidence Intervals From Conformal Inference eFigure 5. Census Tract Log Median Biweekly Spending and Log Median Biweekly Foot Traffic in Study Period eTable 1. Data Sources Used for Study Analyses eTable 2. Augmented Synthetic Control Results for Consumer Spending and Foot Traffic Without Neighborhood Covariates eMethods eReferences [file jamanetwopen-e2559863-s001.pdf]

Supplemental Online Content

Allen B, Basaraba C, Chambers LC, Behrends CN, Marshall BDL, Cerdá M. Overdose prevention centers and neighborhood commercial activity in New York City. *JAMA Netw Open*. 2026;9(2):e2559863. doi:10.1001/jamanetworkopen.2025.59863

eFigure 1. Estimated Effects at Post-OPC Timepoints From Augmented Synthetic Control Models With Neighborhood Covariates[1], 95% Confidence Intervals From Conformal Inference

eFigure 2. Consumer Spending for Treated and Synthetic Neighborhoods From Augmented Synthetic Control Models Without Neighborhood Covariates

eFigure 3. Foot Traffic for Treated and Synthetic Neighborhoods From Augmented Synthetic Control Models Without Neighborhood Covariates

eFigure 4. Estimated Effects at Post-OPC Timepoints From Augmented Synthetic Control Models Without Neighborhood Covariates, 95% Confidence Intervals From Conformal Inference

eFigure 5. Census Tract Log Median Biweekly Spending and Log Median Biweekly Foot Traffic in Study Period

eTable 1. Data Sources Used for Study Analyses

eTable 2. Augmented Synthetic Control Results for Consumer Spending and Foot Traffic Without Neighborhood Covariates

eMethods

eReferences

This supplemental material has been provided by the authors to give readers additional information about their work.

© 2026 Allen B et al. *JAMA Network Open*

eFigure 1. Estimated Effects at Post-OPC Timepoints from Augmented Synthetic Control Models with Neighborhood Covariates[1], 95% Confidence Intervals from Conformal Inference

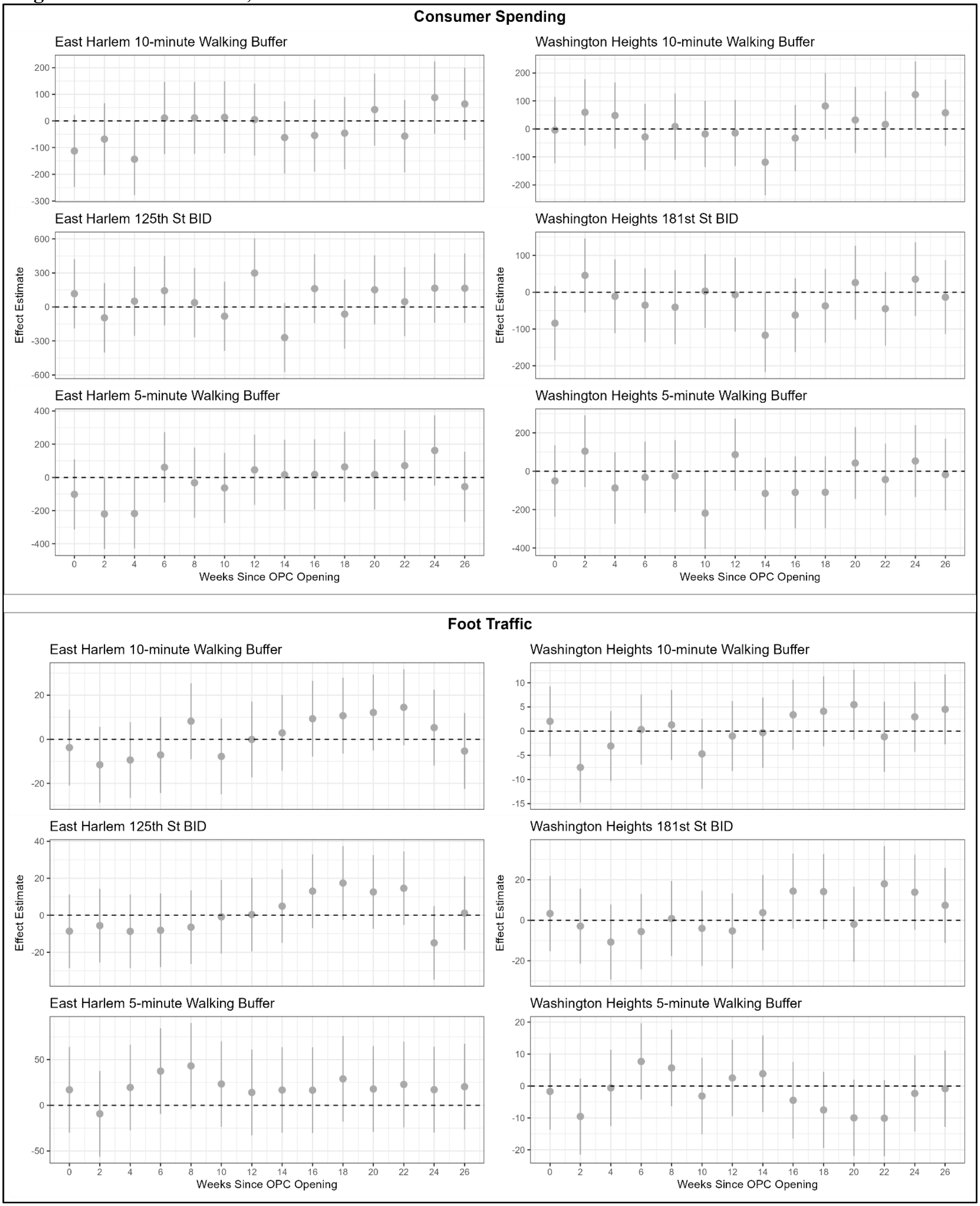

[1]Neighborhood covariates: median age, median household income, proportion of residents unemployed, proportion of families living below the federal poverty line, proportion of individuals 25 or over with a high school diploma, gentrification, proportion of grocery store POI, proportion of restaurant POI, COVID-19 hospitalization rate, and NYPD arrests

eFigure 2. Consumer Spending for Treated and Synthetic Neighborhoods from Augmented Synthetic Control Models without Neighborhood Covariates

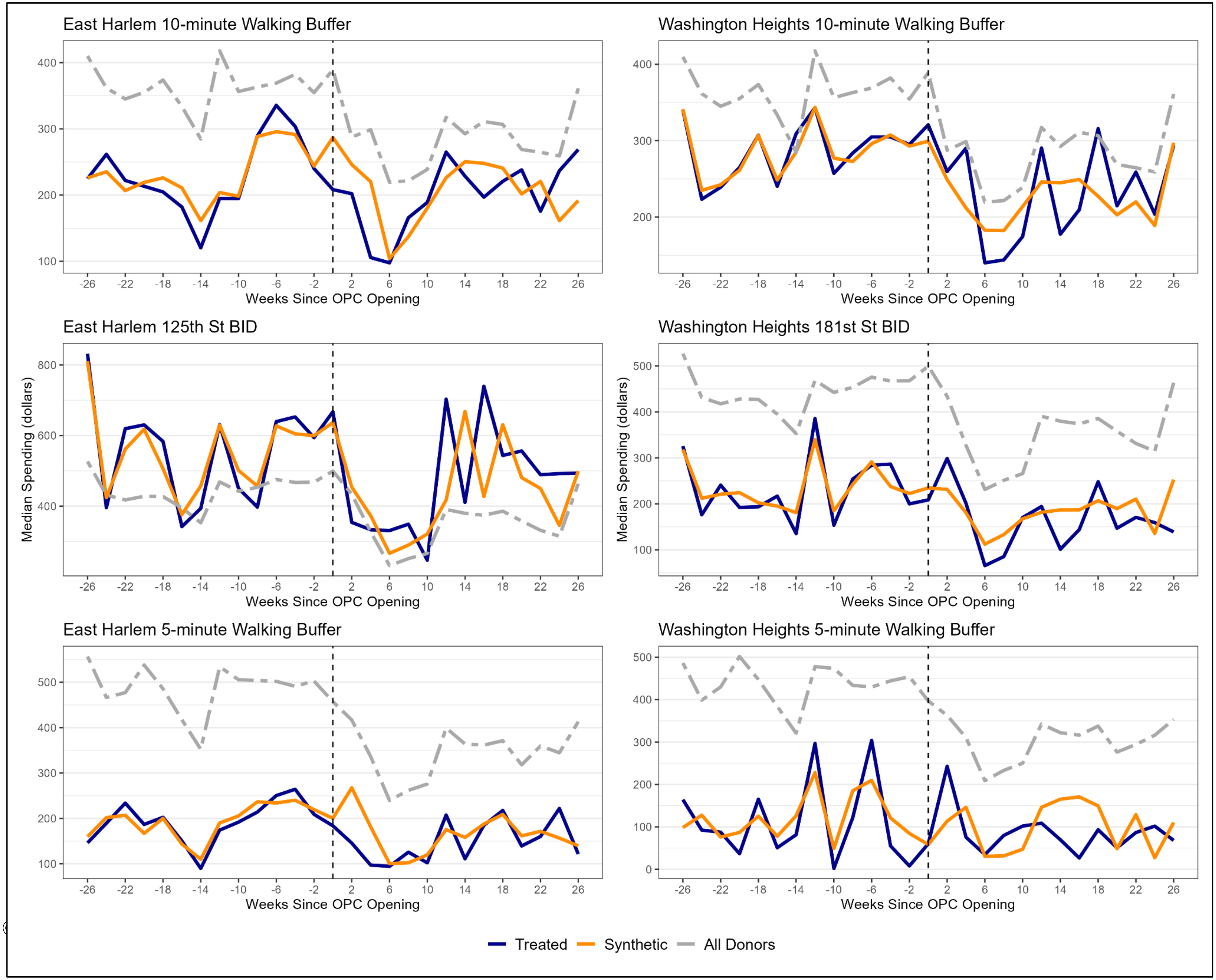

eFigure 3. Foot Traffic for Treated and Synthetic Neighborhoods from Augmented Synthetic Control Models without Neighborhood Covariates

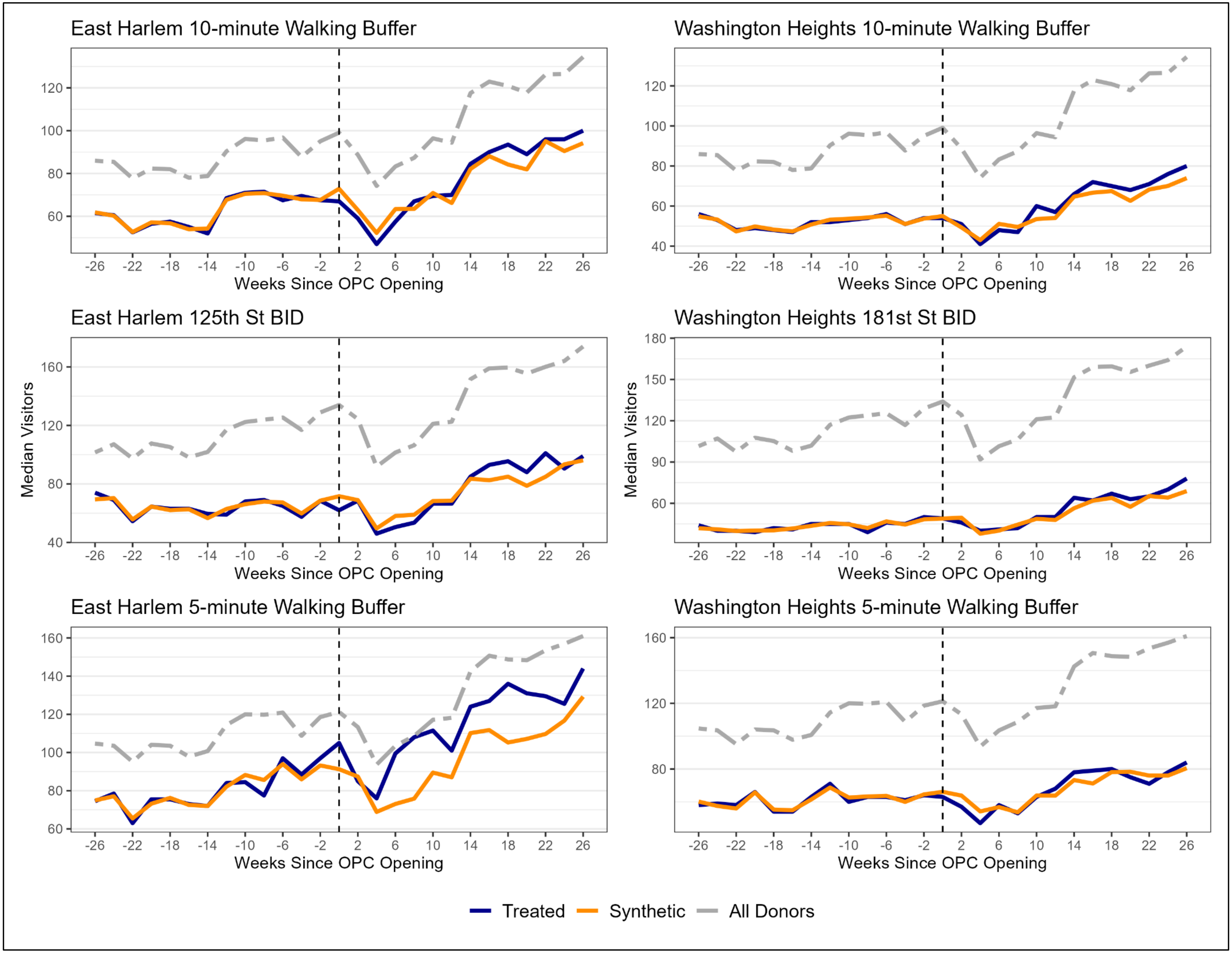

eFigure 4. Estimated Effects at Post-OPC Timepoints from Augmented Synthetic Control Models without Neighborhood Covariates, 95% Confidence Intervals from Conformal Inference

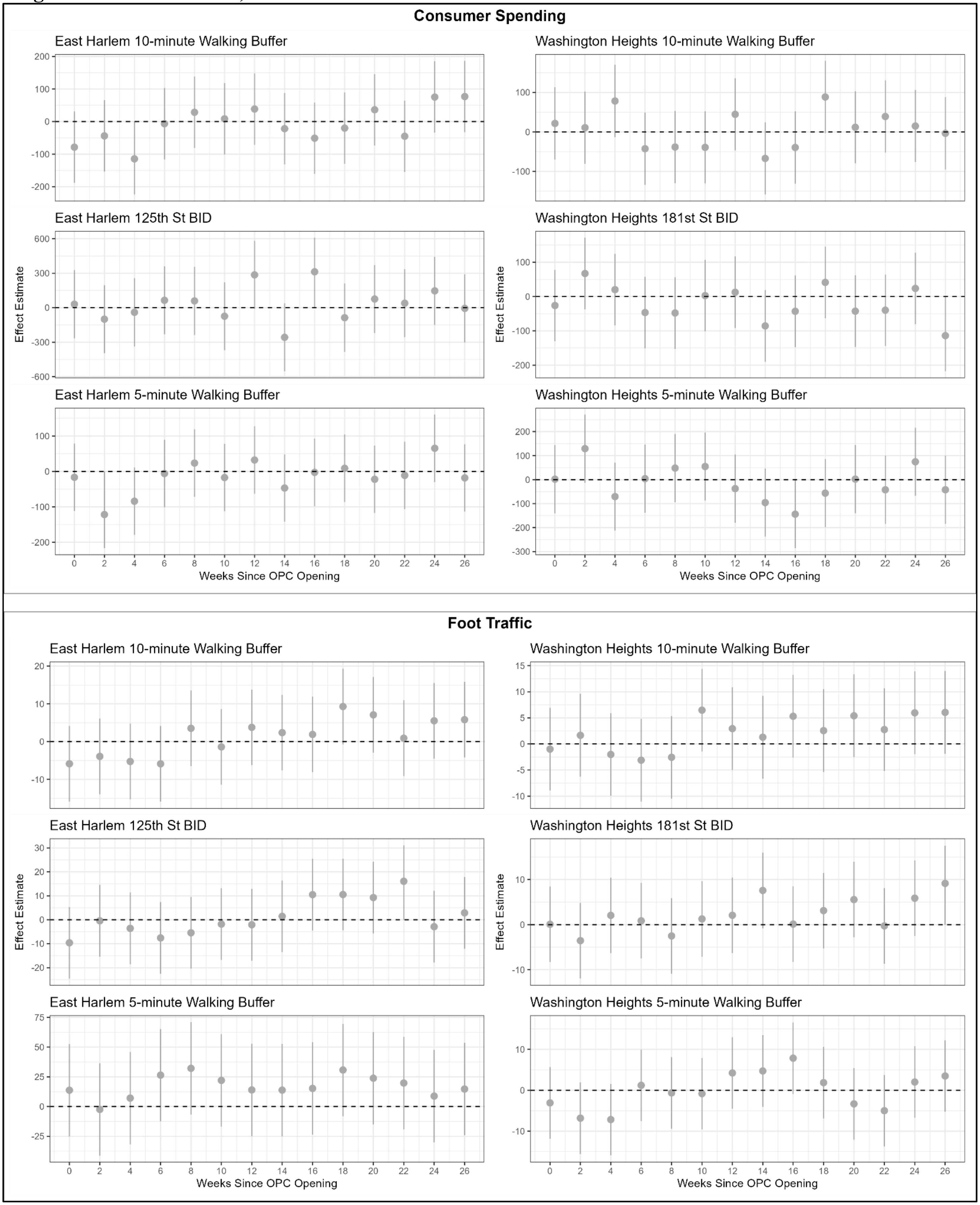

© 2026 Allen B et al. *JAMA Network Open*

eFigure 5. Census Tract Log Median Biweekly Spending and Log Median Biweekly Foot Traffic in Study Period

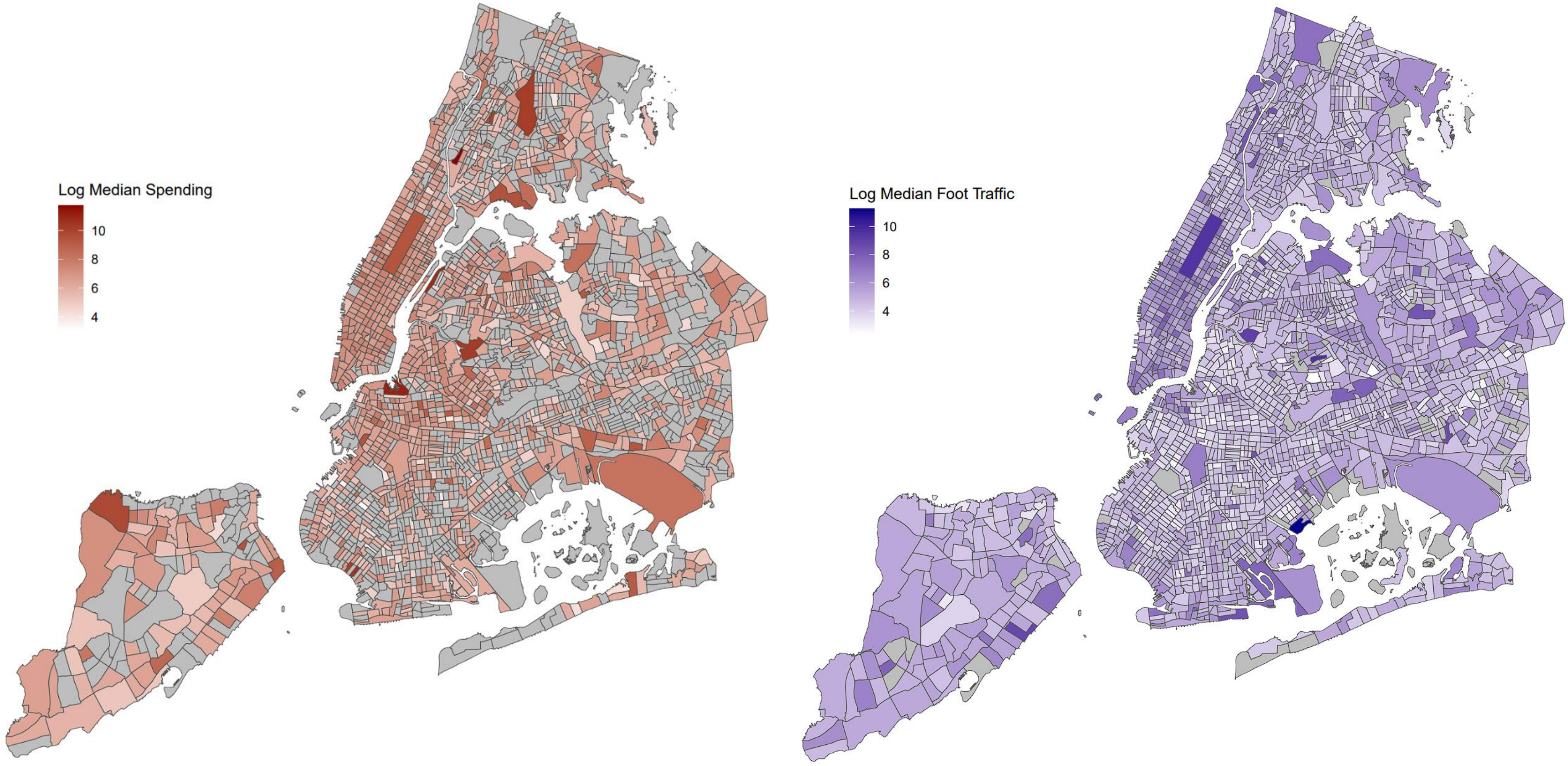

© 2026 Allen B et al. *JAMA Network Open*

eTable 1. Data Sources Used for Study Analyses

Construct	Source	Unit of Observation	Raw Spatial Resolution	Raw Temporal Resolution	Description of Study-Period Harmonization	Use in Analysis
Consumer mobility (foot traffic)	SafeGraph: People and Places (Dewey)	POI	Point (lat/long)	Daily	Aggregated as median visits within each buffer or BID during each biweekly period	Outcome
Consumer spending	SafeGraph: Spend (Dewey)	POI	Point (lat/long)	Daily	Aggregated as median spending within each buffer or BID during each biweekly period	Outcome
POI characteristics: % restaurants; % grocery stores	SafeGraph: People and Places (Dewey)	POI	Point (lat/long)	Static	Aggregated as the percentage of POIs that were classified as restaurants or grocery stores within each buffer or BID	Covariate
Neighborhood sociodemographics: median age; median household income; % unemployed; % families below poverty line; % high school educated	US Census/American Community Survey	Census tract	Census tract	5-year estimate (2017-2021)	Buffer or BID values obtained through areal interpolation of census tract level sociodemographic variable	Covariate
Neighborhood gentrification	US Census/American Community Survey	Census tract	Census tract and core-based statistical area	5-year estimates (2017-2021) and (2007-2011)	Buffer or BID values obtained through areal interpolation of census tract level binary gentrification variable, which was calculated by determining if the change of the ratio of the tract's median income to the median income of its surrounding core-based statistical area was greater than 0.1	Covariate
COVID-19 burden: hospitalization rate per 100,000	New York City Department of Health and Mental Hygiene	ZIP code	ZIP code	Monthly	Buffer or BID values obtained through areal interpolation of ZIP codes in each biweekly period for each biweekly period	Time-varying covariate
Neighborhood crime burden: arrests for any charge	New York Police Department	Arrest	Point (lat/long)	Day	Aggregated as total arrests within each buffer or total arrests per square kilometer for BIDs during each biweekly period	Time-varying covariate

Abbreviations: POI, point of interest; BID, Business Improvement District

© 2026 Allen B et al. *JAMA Network Open*

eTable 2. Augmented Synthetic Control Results for Consumer Spending and Foot Traffic without Neighborhood Covariates

Consumer Spending					
	Pre-OPC RMSE	Post-OPC RMSE	Post/Pre Ratio	Average ATT (SE[1])	p-value[2]
**10-minute Walking Buffer**					
East Harlem	21.0493	54.8210	2.6044	-8.351 (3.25)	0.3214
Washington Heights	10.4122	45.7978	4.3985	5.571 (22.08)	0.1250
**BID**					
East Harlem	43.4293	148.2276	3.4131	32.100 (19.57)	0.1714
Washington Heights	29.7937	52.1216	1.7494	-19.843 (15.73)	0.6714
**5-minute Walking Buffer**					
East Harlem	17.1020	47.5512	2.7805	-15.599 (7.67)	0.4048
Washington Heights	57.1985	70.9728	1.2408	-12.427 (15.96)	0.7000
**Foot Traffic**					
	Pre-OPC RMSE	Post-OPC RMSE	Post/Pre Ratio	Average ATT (SE[1])	p-value[2]
**10-minute Walking Buffer**					
East Harlem	1.1039	5.0120	4.5403	1.285 (1.69)	0.3860
Washington Heights	0.7168	3.9616	5.5271	2.265 (0.76)	0.3158
**BID**					
East Harlem	2.2063	7.4865	3.3932	1.245 (2.62)	0.5540
Washington Heights	1.3921	4.1914	3.0108	2.242 (1.75)	0.6351
**5-minute Walking Buffer**					
East Harlem	3.1316	19.4226	6.2022	17.143 (2.37)	0.2632
Washington Heights	1.5128	4.3700	2.8887	-0.108 (2.27)	0.5789

[1]Jacknife standard errors

[2]Permutation p-values from placebo tests

© 2026 Allen B et al. *JAMA Network Open*

eMethods

SafeGraph Data

The SafeGraph dataset is made up of non-residential points of interest (POIs) that have been identified through web-crawling, publicly available APIs, and licensing third-party data. In addition to geolocation, these data sources were used to obtain detailed information about POIs, including brand affiliation, open hours, North American Industry Classification System (NAICS) codes, and POI category. Proprietary de-duplication and merging processes are performed, as well as modeling and imputation to infer information when it is otherwise unavailable. The spatial geometry of SafeGraph POIs is determined via third-party data sources and machine learning models which incorporate satellite imagery.[1]

The SafeGraph spending dataset aggregates anonymized debit and credit card transactions at a subset of individual SafeGraph POIs via a partnership with one of the largest debit and credit transaction data providers. SafeGraph Spend data is aggregated at the daily level, and does not include information about individual consumers or transactions.[1] This spending dataset was made freely available as part of the COVID-19 Data Consortium,[2] which led to several public studies on the effects of COVID-19 lockdowns on consumer spending.[3-5]

The SafeGraph foot traffic dataset uses GPS location data from mobile phones to attribute visits to non-residential POIs. The details of this attribution process are proprietary, but SafeGraph shares their overall approach, which is to: (1) clean raw GPS data to deal with GPS signal drift, spiking horizontal accuracies, and jumpy GPS pings; (2) cluster GPS pings together using POI data for context as well as a modified density-based spatial clustering of applications with noise (DBSCAN) approach; (3) join identified clusters with spatial footprints of DOIs; and (4) use prediction models to attribute clusters to specific POIs based on contextual factors, such as time and POI category.[6] This foot traffic dataset was also made available as part of the COVID-19 Data Consortium, and has been used in highly cited studies of movement and the effect of public health interventions during the COVID-19 pandemic.[7-9]

For this study, foot traffic data within SafeGraph were obtained through cell phone "pings," which denote unique and repeat walk-in entrances to POIs as a measure of consumer mobility. Consumer spending data captured spending from credit and debit cards for offline and online purchases at POIs; only in-person spending was included in our analyses. POIs with available foot traffic data in our study period we refer to as "foot traffic POIs," and the subset with available spending data we refer to as "consumer spending POIs." We used these data sources to assess changes in consumer mobility patterns into POIs and spending within POIs in the areas surrounding the NYC OPCs before and after the OPCs opened on November 30, 2021.

Construction of Spatial Buffers

We defined the neighborhoods surrounding the OPCs in three ways: (1) 10-minute walking buffers; (2) 5-minute walking buffers; and (3) Business Improvement Districts (BIDs), which are areas in NYC where businesses collaborate to enhance neighborhood economic vitality (Figure 1).

Primarily, we selected the 10-minute walking time buffer to ascertain the impact of the OPCs on commercial activity in their immediate vicinities. We selected a 10-minute walking time guided by prior, NYC-based research showing that SSP client service use increased with travel distances within 1 mile (approximately 20 minutes walking),[10] and that over half of NYC SSP clients, when surveyed, expressed willingness to travel up to 1 mile for SSP services.[11] To

look at businesses and movement patterns more proximally to the OPCs—assuming businesses closer to the OPCs would acutely experience any economic changes—we narrowed that previously established distance to a 10-minute walking buffer.

Secondarily, we selected 5-minute walking buffers to capture a narrower range around each OPC. We also included BIDs as a secondary, policy-relevant geographic unit due to their political and community influence in NYC and proximal locations to each of the two OPCs,[12,13] recognizing that BID boundaries are administratively defined and may not fully overlap with our walking buffers around OPC sites. Further, prior research using SafeGraph mobility data has identified that consumer mobility in dense urban US contexts may be responsive to property and violent crime within bounds of up to 500 meters (approximately 0.3 miles or an approximately 6-minute walking time),[14] suggesting plausible consumer responsiveness to the OPCs within our selected primary and secondary boundaries.

Covariates

We included several potential drivers of foot traffic and consumer spending in our models, specifically: neighborhood socioeconomic conditions derived from the US Census/American Community Survey, SafeGraph POI-level variables, COVID-19 hospitalization rate, and NYPD arrests. Census-derived features of interest included median age, median household income, proportion of residents unemployed, proportion of families living below the federal poverty line, and proportion of individuals 25 or over with a high school diploma. We also calculated a measure of neighborhood gentrification, defining Census tracts within neighborhoods as gentrifying if the change in the ratio of the tract's median income to the median income of its surrounding US Census core-based statistical area was at least 0.1 over the prior 10-year period.[15] To aggregate Census-derived variables at each of our neighborhood definitions, we used areal interpolation that incorporated all census tracts intersecting each BID or buffer. We incorporated SafeGraph POI-level variables by calculating the proportion of POIs that were labeled restaurants and the proportion of POIs that were labeled grocery stores within each defined neighborhood. To reflect the neighborhood crime burden, we used publicly available geocoded NYPD arrest data[16] to construct a covariate representing the number of arrests per square kilometer for BIDs and the total number of arrests in walking buffers for each biweekly period, inclusive of all charge types.

As our study period included the Delta and Omicron waves of the COVID-19 pandemic in NYC,[17] we also added monthly COVID-19 hospitalization rate per 100,000 residents,[18] available at ZIP code resolution, as a covariate in our models. We selected hospitalization rates as a clinically meaningful indicator of area-level COVID-19 burden that is more stable and less sparse than death rates at a small-area level, as well as more systematically documented and less sensitive to neighborhood differences in testing availability than area-level incidence rates or testing rates.[19] As such, we used hospitalization rates as a proxy measure for pandemic intensity that may influence consumer behavior through neighborhood-level public health guidance and health system strain.

Statistical Analysis

We calculated the median and interquartile range of the biweekly median consumer spending and foot traffic measures within each catchment area (10-minute walking buffer, BID, and 5-minute walking buffer) for both OPC neighborhoods (East Harlem and Washington

Heights) and all synthetic control donor units, for the pre- and post-OPC periods. Differences between intervention periods were tested using the Wilcoxon rank-sum test.

We used Augmented Synthetic Controls (ASC)[20] to examine the effect of opening NYC OPCs on median biweekly spending and median biweekly foot traffic in the areas surrounding each OPC. Synthetic control methods (SCM) are used to estimate causal effects of interventions that occur in a small number of treated units, often states or administrative areas, when there is no appropriate control for comparison. SCMs use a weighted combination of untreated units from a donor pool to construct a "synthetic" control unit that closely resembles the outcome behavior of the treated unit prior to the intervention; other covariates are often included to improve comparability between treated and synthetic units across relevant factors.[21,22] If the synthetic control closely matches the treated unit's outcome trajectory in the pre-intervention period, and assuming there is no interference between treated and donor units, no anticipation effect of the intervention, and no time-varying unobserved confounding, then the synthetic control unit provides a valid counterfactual for the treated unit.[21] Consequently, the observed difference between the treated and synthetic units in the post-intervention period represents the average treatment effect on the treated (ATT).

The ASC approach improves on traditional SCM by including a ridge regression outcome model which corrects for imperfect pretreatment fit and allows for optimal covariate balance through residualization.[20] The most common form of significance testing for SCM is placebo-testing, a permutation approach where a SCM is fit for each unit in the donor pool as if it were the treated unit with the same intervention time, and a distribution of "placebo effects" are generated. The Root Mean Squared Error (RMSE) for pre- and post-intervention periods is calculated for the observed and placebo effects, and then a p-value is obtained by rank-ordering the ratio of post- to pre-intervention RMSE.[23] This approach tests for differences across the entire post-intervention period; additional methods, such as a conformal inference procedure,[24] have been developed to test effects at specific timepoints. In this analysis, we report the average biweekly treatment effect over the entire post-intervention period and evaluate the significance of this effect using placebo tests. We also use the conformal inference procedure implemented in the *augsynth* package to construct 95% confidence intervals and evaluate the significance of effects at each post-intervention timepoint.

For our primary analysis, we separately considered the effect of OPC openings in East Harlem and Washington Heights on the 10-minute walking buffer around each OPC. We considered 10-minute walking buffers around SSPs without supervised consumption spaces and opioid treatment programs (OTPs) as our donor pool; specifically, there were 16 SSPs and 39 OTPs with valid SafeGraph spending data and 16 SSPs and 40 OTPs with valid foot traffic data. These donor units contained a total of 7,501 foot traffic POIs and 1,227 consumer spending POIs within 10-minute walking buffers. As a secondary analysis we considered the effect of the East Harlem OPC opening on the 125th Street BID and the Washington Heights OPC opening on the 181st Street BID; we used all other NYC BIDs (n = 74) as a donor pool to construct each synthetic control. There were a total of 8,231 foot traffic POIs and 3,111 spending POIs within donor BIDs, and none of these donor BIDs were within the same community districts as the treated BIDs. Finally, we examined the effect of OPCs within a smaller 5-minute walking buffer. For foot traffic within 5-minutes, we used the same set of donor SSPs and OTPs, with a total of 2,478 foot traffic POIs. However, there were 5-minute donor buffers with very high biweekly spending compared to OPC buffers, which led to poor ASC model fit and unrealistic negative counterfactual spending in the post-OPC period. We therefore limited our donor pool to those

SSP and OTP 5-minute buffers where median biweekly spending in the pre-OPC period was less than or equal to 10 times the median biweekly spending of the treated buffer. This resulted in 14 SSPs and 27 OTPs with a total of 397 spending POIs for the East Harlem analysis, and 14 SSPs and 25 OTPs with a total of 374 spending POIs for the Washington Heights analysis. All analyses and visualizations were performed using R Version 4.4.[25]

© 2026 Allen B et al. *JAMA Network Open*

eReferences

1. SafeGraph's Data Sourcing Process. SafeGraph. Updated 03/26/2021. Accessed 12/8/2025, 2025. https://www.safegraph.com/blog/safegraphs-data-sourcing-process
2. SafeGraph Partners with Dewey to Democratize Access to Data for Academics. Updated 09/02/2022. Accessed 12/08/2025, 2025. https://www.safegraph.com/blog/safegraph-partners-with-dewey
3. Li K, Foutz NZ, Cai Y, Liang Y, Gao S. Impacts of COVID-19 lockdowns and stimulus payments on low-income population's spending in the United States. *PloS one*. 2021;16(9):e0256407.
4. Rose N, Rowe F, Dolega L. How consumer behaviours changed in response to COVID-19 lockdown stringency measures: A case study of Walmart. *Applied Geography*. 2023;154:102948.
5. Yang Z, Choe Y, Martell M. COVID-19 economic policy effects on consumer spending and foot traffic in the US. *Journal of Safety Science and Resilience*. 2021;2(4):230-237.
6. A Technical Guide to SafeGraph Places Data. Accessed 12/08/2025, 2025. https://www.safegraph.com/guides/places-data-technical-guide
7. Chang S, Pierson E, Koh PW, et al. Mobility network models of COVID-19 explain inequities and inform reopening. *Nature*. 2021;589(7840):82-87.
8. Weill JA, Stigler M, Deschenes O, Springborn MR. Social distancing responses to COVID-19 emergency declarations strongly differentiated by income. *Proceedings of the national academy of sciences*. 2020;117(33):19658-19660.
9. Jay J, Bor J, Nsoesie EO, et al. Neighbourhood income and physical distancing during the COVID-19 pandemic in the United States. *Nature human behaviour*. 2020;4(12):1294-1302.
10. Cooper HL, Des Jarlais DC, Ross Z, Tempalski B, Bossak B, Friedman SR. Spatial access to syringe exchange programs and pharmacies selling over-the-counter syringes as predictors of drug injectors' use of sterile syringes. *Am J Public Health*. Jun 2011;101(6):1118-25.
11. Calvo M, MacFarlane J, Zaccaro H, et al. Young people who use drugs engaged in harm reduction programs in New York City: Overdose and other risks. *Drug Alcohol Depend*. Sep 1 2017;178:106-114.
12. Elmedni B, Christian N, Stone C. Business improvement districts (BIDs): An economic development policy or a tool for gentrification. *Cogent Business & Management*. 2018/01/01 2018;5(1):1502241.
13. Armstrong A, Gould Ellen I, Schwartz AE, Voicu I. *The Benefits of Business Improvement Districts: Evidence from New York City*. Furman Center for Real Estate and Urban Policy, NYU Wagner School of Public Service, NYU School of Law; 2007.
14. Fe H, Sanfelice V. How bad is crime for business? Evidence from consumer behavior. *Journal of Urban Economics*. 2022/05/01/ 2022;129:103448.
15. Ellen IG, Torrats-Espinosa G. Gentrification and Fair Housing: Does Gentrification Further Integration? *Housing Policy Debate*. 2019/09/03 2019;29(5):835-851.
16. NYPD Arrests Data (Historic). NYC Open Data. Accessed 03/30, 2025. data.cityofnewyork.us/Public-Safety/NYPD-Arrests-Data-Historic-/8h9b-rp9u/about_data

© 2026 Allen B et al. *JAMA Network Open*

17. Carreño JM, Wagner AL, Monahan B, et al. SARS-CoV-2 serosurvey across multiple waves of the COVID-19 pandemic in New York City between 2020–2023. *Nature communications*. 2024;15(1):5847.
18. COVID-19 data. New York City Department of Health and Mental Hygiene. Accessed 03/30, 2025. https://www1.nyc.gov/site/doh/covid/covid-19-data.page
19. Lieberman-Cribbin W, Tuminello S, Flores RM, Taioli E. Disparities in COVID-19 Testing and Positivity in New York City. *American journal of preventive medicine*. 2020/09/01/ 2020;59(3):326-332.
20. Ben-Michael E, Feller A, Rothstein J. The Augmented Synthetic Control Method. *Journal of the American Statistical Association*. 2021/10/02 2021;116(536):1789-1803.
21. Abadie A. Using synthetic controls: Feasibility, data requirements, and methodological aspects. *Journal of economic literature*. 2021;59(2):391-425.
22. Pickett RE, Hill J, Cowan SK. *The Myths of Synthetic Control: Recommendations for Practice*. 2022.
23. Abadie A, Diamond A, Hainmueller J. Comparative Politics and the Synthetic Control Method. *American Journal of Political Science*. 2015;59(2):495-510.
24. Chernozhukov V, Wüthrich K, Zhu Y. An Exact and Robust Conformal Inference Method for Counterfactual and Synthetic Controls. *Journal of the American Statistical Association*. 2021/10/02 2021;116(536):1849-1864.
25. *R: A Language and Environment for Statistical Computing*. R Foundation for Statistical Computing, Vienna, Austria; 2025.

© 2026 Allen B et al. *JAMA Network Open*
