# Supplementary material for: Overdose Prevention Centers and Neighborhood Commercial Activity in New York City
Source: JAMA Netw Open. 2026 Feb 27;9(2):e2559863. doi: 10.1001/jamanetworkopen.2025.59863 (PMC12949448; doi:10.1001/jamanetworkopen.2025.59863)
Supplement: Supplement 2. — Data Sharing Statement [file jamanetwopen-e2559863-s002.pdf]

Data Sharing Statement

Allen. Overdose Prevention Centers and Neighborhood Commercial Activity in New York City. *JAMA Netw Open*. Published February 27, 2026. doi:10.1001/jamanetworkopen.2025.59863

Data
Data available: No

Additional Information
Explanation for why data not available: Data used for this study are available through institutional data use agreements with Dewey Data Systems and cannot be shared publicly. Analytic code is available at: https://github.com/nyu-spotlight/opc-neighborhood-safegraph.
